# Transcriptome- and Epigenome-Wide Association Studies of Tic Spectrum Disorder in Discordant Monozygotic Twins

**DOI:** 10.3390/genes17010097

**Published:** 2026-01-18

**Authors:** Jonas Dalsberg, Cathrine Jespersgaard, Amanda M. Levy, Anna Maria Asplund, Frederik Otzen Bagger, Nanette M. Debes, Qihua Tan, Zeynep Tümer, Mathis Hildonen

**Affiliations:** 1 Department of Clinical Genetics, Copenhagen University Hospital, Rigshospitalet, 2100 Copenhagen, Denmark; jonas.dalsberg.joergensen@regionh.dk (J.D.); cjespersgaard@hotmail.com (C.J.); marie.amanda.levy@regionh.dk (A.M.L.); anna.maria.asplund@regionh.dk (A.M.A.); mathis.hildonen@regionh.dk (M.H.); 2 Department of Genomic Medicine, Copenhagen University Hospital, Rigshospitalet, 2100 Copenhagen, Denmark; frederik.otzen.bagger@regionh.dk; 3 National Tourette Clinic, Department of Pediatrics, Copenhagen University Hospital, 2730 Herlev, Denmark; nanette.marinette.monique.debes@regionh.dk; 4 Department of Clinical Medicine, Faculty of Health and Medical Sciences, University of Copenhagen, 2200 Copenhagen, Denmark; 5 Research Unit for Epidemiology, Biostatistics and Biodemography, Department of Public Health, University of Southern Denmark, 5230 Odense, Denmark; qtan@health.sdu.dk; 6 Unit of Human Genetics, Department of Clinical Research, University of Southern Denmark, 5230 Odense, Denmark

**Keywords:** DNA methylation, epigenetics, gene expression, GTS, tic spectrum disorder, tics, Tourette syndrome, RNA sequencing

## Abstract

**Background**: Tic spectrum disorder (TSD), encompassing Tourette syndrome and chronic tic disorder, is a childhood-onset neurodevelopmental condition with complex genetic and environmental contributions. Heritable components have been implicated in TSD, but no clear genetic mechanisms have been identified. Significant aspects of TSD etiology remain unclear, with key uncertainties concerning the role of environmental influences in its development. In this study, we aimed to identify environmentally induced epigenomic and transcriptomic changes contributing to TSD pathology by investigating genetically similar monozygotic twins discordant for TSD. **Methods**: To investigate environmentally driven mechanisms, we analyzed peripheral blood from eleven monozygotic twin pairs, either discordant or concordant for TSD, using RNA sequencing and DNA methylation analysis. **Results**: Differential expression analysis identified a dozen differentially expressed genes between TSD and non-TSD individuals, most of which were long non-coding RNAs or pseudogenes. Expression of the small RNA gene *RNY1* was significantly associated with tic severity, suggesting involvement of immune-related processes. DNA methylation (DNAm) analysis revealed ~30,000 probes with a nominal *p* < 0.05, however none of these were significant after multiple testing correction. Expression quantitative trait methylation (eQTM) analysis identified 236 methylation-associated genes. Gene set enrichment analysis demonstrated broad downregulation in TSD individuals for pathways related to translation, RNA processing, and neurobiological functions, with Kyoto Encyclopedia of Genes and Genomes (KEGG) pathways including ribosome, nucleocytoplasmic transport, pluripotency signaling, and nicotine addiction. **Conclusions**: These results suggest that environmentally influenced gene expression may contribute to TSD pathogenesis through dysregulation of immune and neuronal pathways. Despite a small sample size, the monozygotic twin design provides strong control for genetic background and identifies significant differences that contribute to the understanding of the underlying molecular mechanisms of TSD.

## 1. Introduction

Tourette syndrome (TS) is a childhood-onset multifactorial disorder characterized by motor and vocal tics [1,2,3]. Chronic tic disorder (CTD) differs from TS in that patients have either vocal or motor tics. As no clinical or genetic evidence exists to suggest that TS and CTD are distinct disorders, the term tic spectrum (TSD) disorder has been proposed to cover both [4].

Initial symptom presentation typically occurs between the ages of 3 and 8 years, starting with simple motor tics followed by phonic tics. Symptom severity commonly peaks at ages 8 to 12 years, and by adulthood, most patients will experience symptom attenuation or even complete remission [1,3]. In a recent longitudinal follow-up study of 314 individuals with TSD, only 18% of patients experienced a complete remission after the age of 16 years; 60% still experienced some mild to moderate tics; and the remaining 23% had moderate to severe tics [5]. The reported prevalence of TSD varies from 0.1 to 6% with a male-to-female ratio of 4:1 [3,6]. Most individuals with TSD have comorbidities, of which the most frequent are obsessive–compulsive disorder (OCD) and attention-deficit hyperactivity disorder (ADHD), with as many as 70% of individuals affected by TSD having either disorder. Other common comorbidities include autism spectrum disorder (ASD), anxiety, depression, sleep disorders, migraine, and self-injurious behavior [1,3].

Several studies have implicated genetics in the etiology of TSD as well as its comorbidities [7,8,9], with large population-based and genome-wide association studies (GWAS) suggesting a heritability of TSD between 0.21 and 0.77 [10,11], where most of the genetic heritability could be explained by evolutionarily conserved SNPs [11]. Various genes and genetic loci have been linked to TSD, but the exact mechanisms underlying the syndrome remain unclear [8]. Genome-wide linkage studies (GWLSs) have identified various genetic loci with potential involvement in TSD etiology, including the two genes Slit and Trk-like 1 (*SLITRK1*) and histidine decarboxylase (*HDC*) [12,13], but reproducibility has been unstable [12,13,14,15]. The dopamine and serotonin systems have also been suggested to be involved in TSD etiology, as drugs inhibiting these neurotransmission systems have been found to reduce tic frequency [16]. Numerous candidate gene studies have been carried out with promising results, but the findings have been irreproducible [8]. Additionally, changes in DNA methylation patterns have been observed in both ADHD and OCD [17,18]. Few methylation studies have been conducted on TSD, but the current findings reveal both overlapping and conflicting results, underscoring the need for further studies [19,20,21]. Finally, the concordance rate for TSD has been reported as 8% and 53% for dizygotic and monozygotic twins, respectively, suggesting that the etiology is not solely determined by genetic components [9]. Instead, TSD seems to have a multifactorial etiology comprising a combination of genetic predisposition and environmental triggers leading to neurobiological vulnerability [22].

Despite advances in research, many gaps in knowledge regarding TSD etiology remain, including but not limited to understanding the environmental contribution to the development of the disorder. In this study, we aimed to identify environmentally induced epigenomic and transcriptomic changes contributing to TSD pathology by investigating genetically similar monozygotic twins discordant for TSD. The monozygotic twin design was expected to ensure minimal genetic variation and, thus, permit the detection of environmentally driven genetic regulation.

## 2. Materials and Methods

### 2.1. Monozygotic Twin TSD Cohort

Cross-linking of The Danish Twin Register and The Danish Psychiatric Central Register with subsequent genetic testing and interview-based diagnosis using DSM-5 led to the identification of fourteen monozygotic twin pairs where at least one twin had TSD, as previously described [23], resulting in a final cohort consisting of five twins discordant for TSD, four twins concordant for TSD, and two asymptomatic twins (Table S1 and Figure S1).

### 2.2. RNA Sequencing

Total RNA was isolated from PAXgene tubes. RNA sequencing was performed using TruSeq Stranded Total RNA Library Prep Kit (Illumina, San Diego, CA, USA), and libraries were paired-end sequenced (2 × 125 bp) on a HiSeq 2500 sequencing system (Illumina).

Reads were mapped to GRCh38.p13 (GCA_000001405.28) using STAR [24]. The resulting count data served as input for downstream pre-processing and differential expression analysis using the edgeR (v. 4.4.2) [25] and limma (v. 3.62.2) [26] R packages. Raw counts were transformed into counts per million (CPM) and log-transformed (LCPM); genes with low expression were filtered using the filterByExpr function; and samples were normalized using the calcNormFactors function from the edgeR package with default settings. Heteroscedasticity was accounted for using voom from limma.

### 2.3. DNA Methylation

Genomic DNA was extracted from peripheral blood and subsequently subjected to bisulfite treatment using standard protocols. Bisulfite-converted DNA was analyzed on Infinium MethlationEPIC v1 arrays (Illumina) according to the manufacturer’s instructions. The data was analyzed using the R package minfi [27]. Exclusion criteria for probes were detection *p*-values > 0.01, SNPs at CpG sites, known cross-reactivity, and location on sex chromosomes. Samples were normalized using stratified quantile normalization. Counts were converted to β values (*β* = *M/M* + *U)* and M-values (*log*2*(M/U)*), where *M* is the methylated intensity, and *U* is the unmethylated intensity. β values were used for methylation quantification, and M-values were used for statistical analyses.

### 2.4. Identification of Differentially Expressed Genes and Differentially Methylated Probes

For both gene expression (RNA sequencing) and DNA methylation data, biological and technical sources of variation were identified using principal component analysis (PCA), and quantile–quantile plots were used to assess differences between TSD and non-TSD individuals as well as to assess deviations from expected data trends and, thus, potential needs for model adjustment. To adjust for unwanted variation in the data, such as cell-type composition, batch effects, and library size, surrogate variables were identified and estimated using the sva R package [28].

Linear models were fitted using limma to assess differences between TSD and non-TSD individuals and to identify associations with Yale Global Tic Severity Scale (YGTSS) scores, incorporating monozygotic twin pairs and surrogate variables as covariates. Correction for multiple testing was performed using the Benjamini–Hochberg method [29]. Two models were fitted for RNA-seq data; one compared TSD cases and controls, and the other tested the association of gene expression levels with YGTSS scores for all samples with YGTSS scores > 0, including concordant twins and asymptomatic controls. For the DNA methylation data, a single model was fitted to compare TSD cases and controls.

### 2.5. Expression Quantitative Trait Methylation (eQTM)

Expression quantitative trait methylation (eQTM) analysis was performed using the R package MatrixEQTL [30]. Gene expression input from RNA sequencing was represented as log2-transformed CPM values with an unadjusted *p*-value threshold of <0.05. For DNA methylation, M-values for probes with unadjusted *p*-values < 0.005 were used. The analysis used a simple t-test and a linear (additive) model adjusted for monozygotic twin pair status. The *p*-value threshold for gene–CpG pairs was set to 0.05, and the analysis was run in cis with a maximum distance of 1 × 10^6^ base pairs.

### 2.6. Gene Set Enrichment Analysis (GSEA)

Gene set enrichment analysis (GSEA) was conducted on the results from the differential expression analysis using the clusterProfiler R package [31] to identify Gene Ontology (GO) and Kyoto Encyclopedia of Genes and Genomes (KEGG) terms associated with TSD. Log2-transformed fold changes were used as input. The geneSet size range was set to 3–800; all subontologies were included; the p-value threshold was set to 0.05; correction for multiple testing was carried out using the Benjamini–Hochberg method; the seed parameter was set to TRUE; and the fast gene enrichment analysis was applied [32].

## 3. Results

### 3.1. Data Exploration and Statistical Model Generation

Principal component analyses (PCAs) were carried out to explore technical variability in the RNA sequencing and DNA methylation data (Figures S2A and S3A), and quantile–quantile (Q-Q) plots were used to assess how the results of the linear models matched the expected null distribution and to identify biases in the results (Figures S2B and S3B). When adjusted for surrogate variables, a close approximation of linearity was achieved in the RNA sequencing data results (Figure S2B). The DNA methylation data showed a linear but deflated distribution of *p*-values, which was partially corrected after surrogate variable adjustment (Figure S3B).

### 3.2. Differential Expression and Methylation Analyses

**Figure 1:**
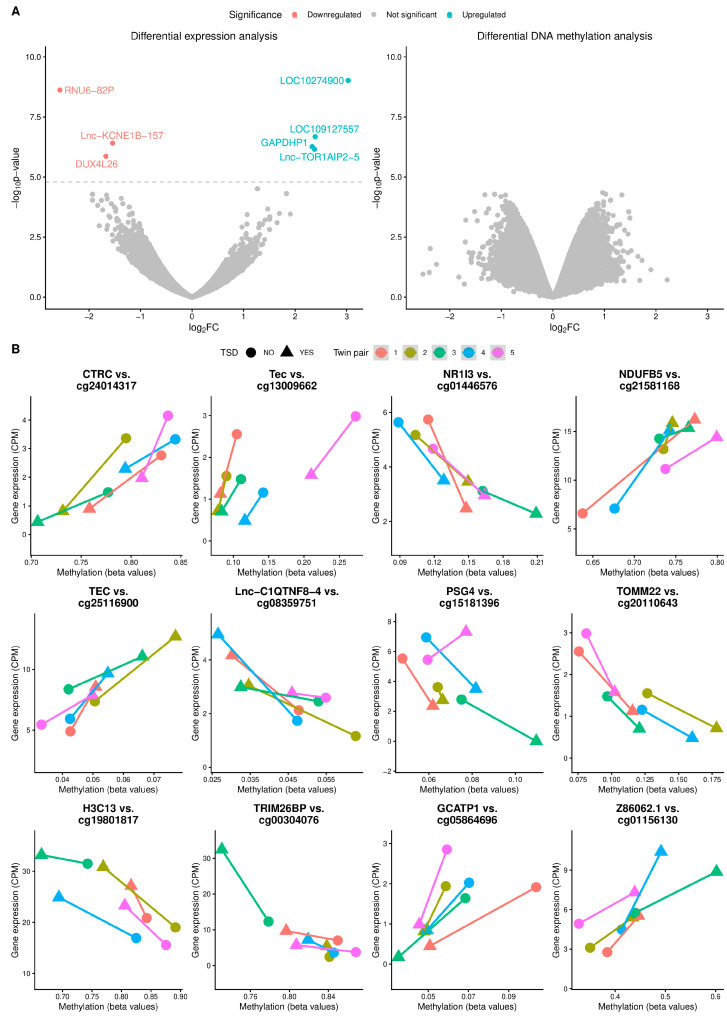
Analysis of RNA sequencing and DNA methylation data. (**A**) Volcano plots showing negative log10-transformed *p*-values (*y*-axis) and log2-transformed fold changes per gene/probe (*x*-axis) for RNA-sequencing (**left**) and DNA methylation (**right**). Significant findings are highlighted. (**B**) Pairwise correlation plots showing gene expression versus methylation levels for the top 12 results from the expression quantitative trait methylation (eQTM) analysis. Each twin pair is represented with a distinct color, and the TSD twin is shown as a triangle, while the asymptomatic twin is indicated by a filled circle.

Differential expression and methylation analyses were carried out to identify genes and probes involved in TSD. Seven genes were found to be differentially expressed in TSD compared to controls (FDR < 0.05, Figure 1A and Table S2). The fold changes of the seven genes ranged between a threefold and an eightfold change in expression (Table S2). When a less stringent FDR was applied (<0.2), a total of 15 DEGs were detected (Table S2). Gene annotation for the 15 genes with FDR < 0.2 is shown in Table S3. None of the DNA methylation probes reached genome-wide significance (Figure 1A); however, 29,868 probes had *p*-values < 0.05, and 1981 probes had *p*-values < 0.005. The expression of one gene, *RNY1*, was found to be significantly associated with the YGTSS score (FDR < 0.05, Table S4).

### 3.3. Expression Quantitative Trait Methylation (eQTM) Analysis

To identify methylation-regulated DEGs in TSD, an expression quantitative trait methylation (eQTM) analysis was performed based on LCPM and M-values. A total of 1201 genes (unadjusted *p* < 0.05) and 1981 probes (unadjusted *p* < 0.005) were included in the analysis. The eQTM analysis resulted in the identification of 236 potentially methylation-regulated genes with *p*-values < 0.05 (Table S6). The top 20 eQTMs are characterized in Table S7.

Gene expression and DNA methylation levels for the top 12 most significant eQTMs are visualized in Figure 1B. All eQTMs plotted had an unadjusted *p*-value < 2.5 × 10^−4^. In all cases except one, the correlation between gene expression and DNA methylation was observed in all samples, and the average methylation changes ranged from 1.8% to 10.0% (Figure 1B).

### 3.4. Gene Set Enrichment Analysis (GSEA)

**Figure 2:**
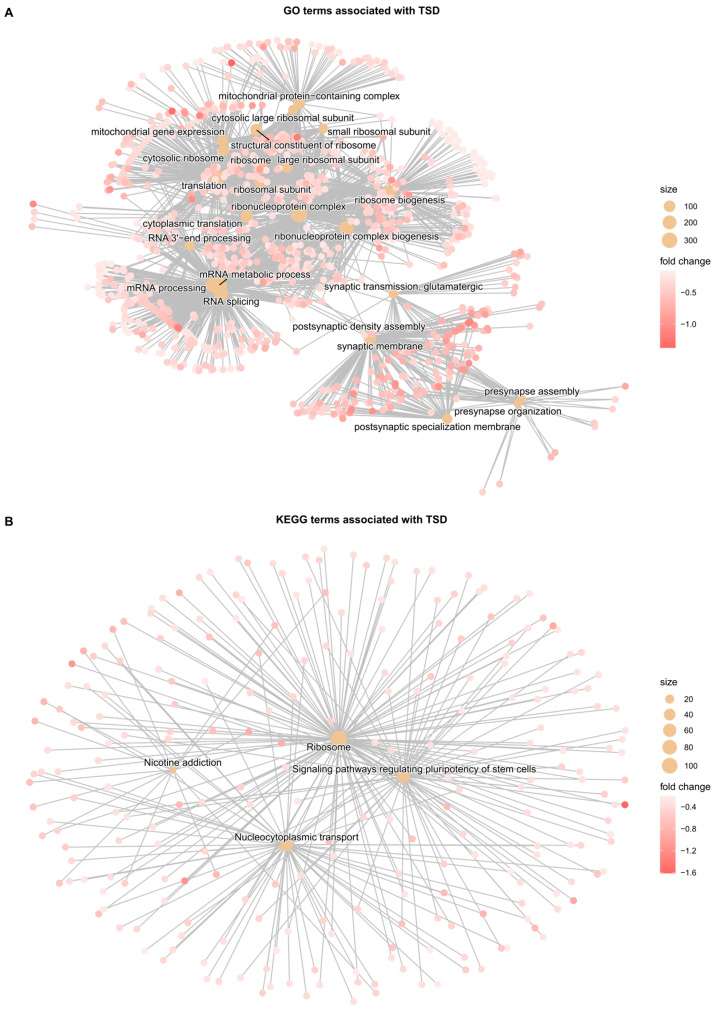
Category net plots for the terms identified in the gene set enrichment analysis for the case–control study. Genes are shown as red dots; fold change is indicated by color intensity; and the number of genes associated with a term is shown as the size of the beige dot. (**A**) Gene Ontology (GO) terms. (**B**) KEGG terms.

Gene set enrichment analysis (GSEA) was conducted to identify differentially regulated ontologies and pathways in TSD compared to non-TSD individuals. We identified 24 GO terms associated with TSD, of which 75% were pathways related to translation and mRNA processing, while 25% were neurological GO terms (Table S8). As shown in the category net plot in Figure 2A, all the significantly altered pathways were downregulated, indicating that the effect on gene expression for TSD was mainly repressive. Regarding KEGG terms, four terms were associated significantly with TSD (Figure 2B)—ribosome, nicotine addiction, nucleocytoplasmic transport, and signaling pathways regulating pluripotency of stem cells. If correction for multiple testing was omitted, 26 KEGG terms were identified (Table S9).

## 4. Discussion

In the present study, we included eleven twins (five twins discordant for TSD, four twins concordant for TSD, and two asymptomatic twins) and analyzed gene expression and DNA methylation data from five monozygotic twin pairs discordant for TSD. Studying discordant monozygotic twins allows us to investigate environmental or other non-genetic factors underlying disease susceptibility, as these individuals share nearly identical genetic backgrounds. We identified seven DEGs with FDR < 5% and 15 DEGs with FDR < 20% (Figure 1A, Table S2). Of the 15 DEGs identified, 11 were lncRNAs and pseudogenes, and most of them were poorly characterized at the time of writing (Table S3) [33,34,35]. To complement the TSD/non-TSD analysis, we investigated associations between gene expression and YGTSS score in 13 individuals affected by TSD. From the top 20 DEGs associated with the YGTSS score, ten genes were involved in regulating immune responses [36,37,38,39,40,41,42,43,44,45,46,47,48,49,50,51,52], and four could be linked to neurological phenotypes [53,54,55,56,57]. Annotation for the YGTSS-associated DEGs can be found in Table S5 [34,58,59,60,61,62,63,64,65,66,67,68,69,70,71,72,73,74,75,76,77,78]. Immune abnormalities have been suggested in the pathogenesis of several movement and psychiatric disorders [79]. Association between immune-based mechanisms and tic exacerbation has been suggested [80]. Furthermore, an association between TSD and autoimmune disorders has previously been reported [3,81,82], and first-degree relatives to TSD patients have been shown to have an increased prevalence of autoimmune disorders such as systemic lupus erythematous (SLE) [81]. SLE patients are also 10–15 times more likely to have OCD, a common comorbidity of TSD [82]. The only gene that achieved genome-wide significance in the YGTSS analysis was *RNY1*, where an inverse relationship between *RNY1* expression and YGTSS score was observed. *RNY1 is* a regulator of the protein Ro60, which is a common autoantigen in both SLE and Sjögren’s syndrome [36]. The earliest detectable autoantibodies in SLE are against Ro60, and autoantibodies precede symptoms, making it a clinically important biomarker [36]. Ro60 seems to have a protective effect against SLE, as Ro60 knockout mice models develop an SLE-like syndrome [83]. As *RNY1* assists Ro60 in its function, depletion of *RNY1* might attenuate the protective effect of Ro60 and be an indication of autoimmunity. Further studies with larger cohorts are necessary to validate this hypothesis. Among the top twenty most significant DEGs associated with YGTSS, we identified *RNU4-2*, *BATF2*, *DUX4L26*, and *MIR4718*, all of which have been associated with neurodevelopmental or neurodegenerative phenotypes [55,56,57,84]. The DEG RP42 Homolog (RP42) pseudogene (*RP1-121G13.3*) has been linked to ADHD, which is a common comorbidity for TSD patients [85] (Supplementary Table S7).

To identify DEGs regulated in cis by DNA methylation, we performed an eQTM analysis, from which we identified 12 cis eQTMs (Figure 1B). The most significant expression–methylation association was between chymotrypsin C (*CTRC*) and cg24014317. Chymotrypsin C is a protease with chymotrypsin-like specificity. This enzyme is also involved in calcium homeostasis and regulation of digestive enzymes such as trypsin and some carboxypeptidases [86,87]. Like its isozyme chymotrypsin, it is highly and almost exclusively expressed in the pancreas. Studies have shown that 60–65% of individuals with ASD have low levels of chymotrypsin, as well as low levels of circulating amino acids, suggesting that increased protease activity might be beneficial [88,89,90]. Pancreatic replacement therapy has recently been investigated as a treatment for ASD [91].

Finally, we identified dysregulated gene ontologies and pathways through gene set enrichment analysis (Figure 2). Most of the detected GO terms were related to translation and mRNA processing, with a select few being related to neurological processes, as one might expect from a neurological disorder (Supplementary Table S8). The identified KEGG terms were related to translation, general cell biology, stem cells, and addiction (Supplementary Table S9). The latter may reflect the ADHD comorbidity, as between 20% and 50% of individuals with ADHD have been reported to have a substance-use disorder [92,93]. Interestingly, all genes belonging to the significantly detected GO or KEGG terms were downregulated in TSD patients relative to controls, suggesting an overall gene-repressive effect in the disorder.

These results suggest that environmentally influenced gene expression may contribute to TSD pathogenesis through dysregulation of immune and neuronal pathways. A limitation of the present study was its small sample size, although the monozygotic twin setup was designed to increase strength by limiting non-disease-specific genetic variation and allowing for the identification of causative factors rooted in environmentally induced changes in gene expression and DNA methylation. Our study design, thus, ensured an almost identical genetic background, such that significant changes in gene expression and/or DNA methylation could be attributed to environmental risk factors.

## Data Availability

The raw data generated in this study cannot be made public due to ethical and legal considerations.

## Supplementary Materials

The following supporting information can be downloaded at https://www.mdpi.com/article/10.3390/genes17010097/s1. Table S1. Metadata table containing patient and sample information. Table S2. Differential expression analysis results table containing analysis results for the 26,618 genes tested for the discordant (TSD vs. non-TSD) monozygotic twins. Table S3. Differential expression analysis annotation table containing gene annotation information on the top 15 most significantly dysregulated genes (TSD vs. non-TSD). All information has been drawn from www.genecards.org and www.gtexportal.org unless a direct link to a different source has been provided in the table. Table S4. Differential expression analysis results table containing analysis results for the 27,262 genes tested for the YGTSS analysis. Table S5. Differential expression analysis annotation table containing gene annotation information on the top 20 most YGTSS-associated genes. All information has been drawn from www.genecards.org and www.gtexportal.org unless a direct link to a different source has been provided in the table. Table S6. Expression quantitative trait loci (eQTL) results table containing analysis results for the 236 gene–probe associations with unadjusted *p*-values below 0.05. Table S7. Expression quantitative trait loci (eQTL) annotation table containing gene annotation information on the top 20 most significant associations. All information has been drawn from www.genecards.org and www.gtexportal.org unless a direct link to a different source has been provided in the table. Table S8. Gene set enrichment analysis (GSEA) table containing analysis results for the 1138 GO terms with unadjusted *p*-values below 0.05. The results are based on a single iteration with the seed parameter set to TRUE in the gseGO() function. Table S9. Gene set enrichment analysis (GSEA) table containing analysis results for the 29 KEGG terms with unadjusted *p*-values below 0.05. The results are based on a single iteration with the seed parameter set to TRUE in the gseKEGG() function. Figure S1. Study design. Twenty-two monozygotic twins participated in this study, including five pairs of twins discordant for TSD, four pairs concordant for TSD, and two asymptomatic pairs. Figure S2. Quality control and data exploration for the RNA sequencing data. (A) Principal component analysis revealing trends in the data. In the top plot, the samples are labeled by sex (M = male; F = female) and colored by library size. In the bottom plot, samples are labeled by twin set (Table S1) and colored by TSD status. PC1 accounts for 55% of the total variance and is mainly driven by differences in library size. The second principal component accounts for 14% of the total variance and seems to be driven solely by sex differences. (B) Quantile–quantile plots of *p*-values from linear models with different adjustments—unadjusted (left) and adjusted for surrogate variables (right). The results from the unadjusted model deviated from linearity. A close approximation of linearity without *p*-value deflation was achieved when the model was adjusted for surrogate variables, where unwanted sources of variation were removed to amplify target differences (TSD vs. non-TSD). Figure S3. Quality control and data exploration for the DNA methylation array data. (A) Principal component analysis revealing trends in the data. In the left plot, the samples are labeled by batch and colored by sex. In the right plot, they are labeled by twin set and colored by TSD status. PC1 accounts for 27% of the total variance and appears to be driven by batch effect, with sex differences seemingly being responsible for the 24% variance represented by PC2. (B) Quantile–quantile plots of *p*-values for different adjustment settings—unadjusted (left) and adjusted for surrogate variables (right). Due to the degrees of freedom requirement for linear models and the low sample size of the present study, it was not possible to include batch effect as a categorical covariate in the model. Instead, a surrogate variable analysis was conducted to correct for the batch effect and any other unwanted sources of variation. The quantile–quantile plots are very linear with a slight *p*-value deflation, possibly because the differences between monozygotic twins are smaller than one would expect at random, and the effect size of TSD is small. This deflation is slightly corrected after surrogate variable adjustment.

## Abbreviations

Abbreviation	Meaning
ADHD	Attention-deficit hyperactivity disorder
ASD	Autism spectrum disorder
CPM	Counts per million
CTD	Chronic tic disorder
DEG	Differentially expressed gene
DMP	Differentially methylated probe
DNAm	DNA methylation
DSM-5	Diagnostic and Statistical Manual of Mental Disorders, Fifth Edition
eQTM	Expression quantitative trait methylation
FDR	False discovery rate
GO	Gene ontology
GSEA	Gene set enrichment analysis
GTS	Gilles de la Tourette syndrome
KEGG	Kyoto Encyclopedia of Gens and Genomes
LCPM	Log-transformed counts per million
OCD	Obsessive–compulsive disorder
PCA	Principal component analysis
Q-Q	Quantile–quantile
SNP	Single-nucleotide polymorphism
TS	Tourette syndrome
TSD	Tic spectrum disorder
YGTSS	Yale Global Tic Severity Scale
