# Supplementary figures and images for: Transcriptome- and Epigenome-Wide Association Studies of Tic Spectrum Disorder in Discordant Monozygotic Twins
Source: Genes (Basel). 2026 Jan 18;17(1):97. doi: 10.3390/genes17010097 (PMC12840948; doi:10.3390/genes17010097)

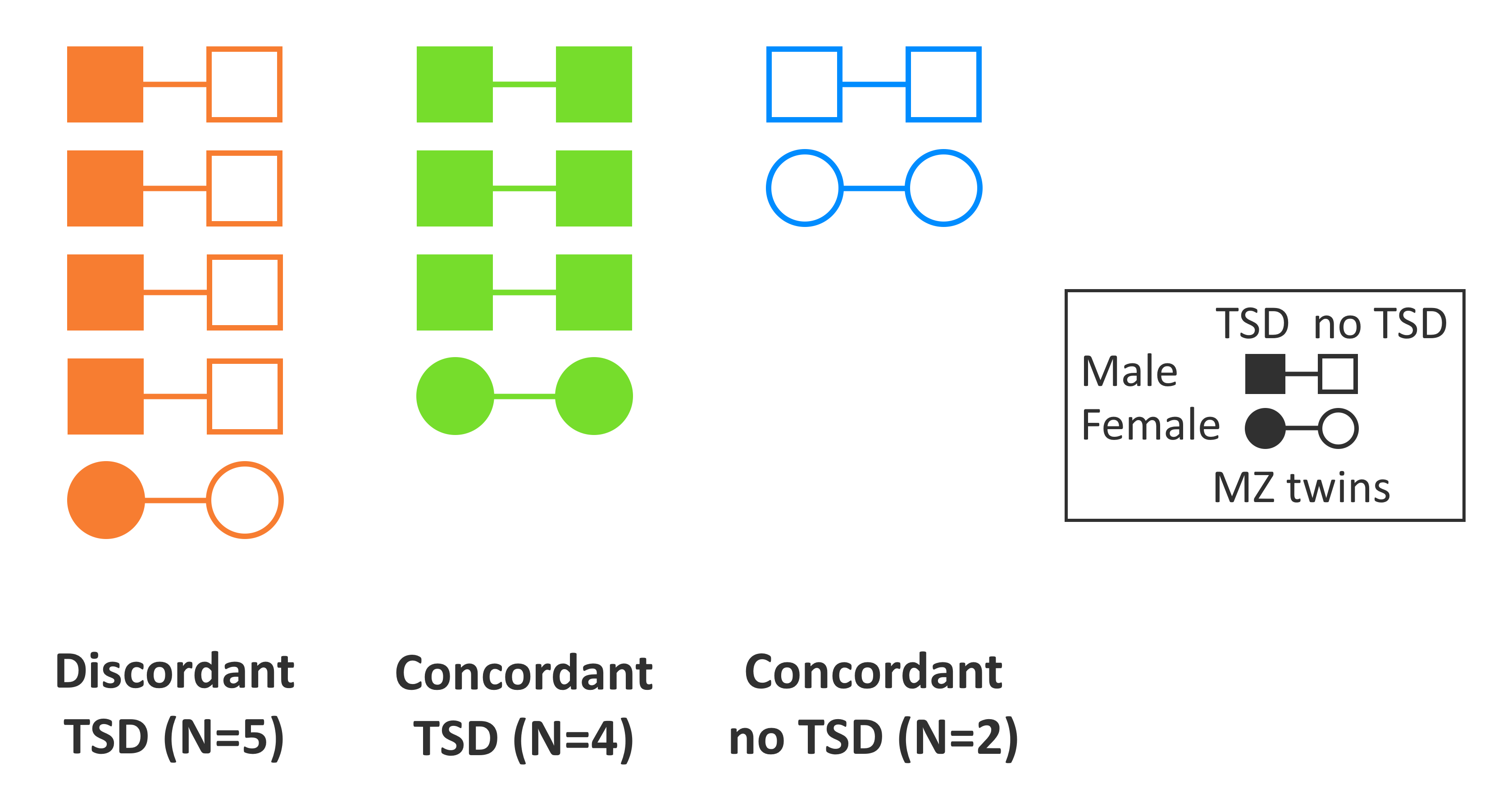

Supplement: Supplementary file 1 [file genes-17-00097-s001.zip › sup_fig_1.png]
